# Changes in gene expression of *Prymnesium parvum* induced by nitrogen and phosphorus limitation

**DOI:** 10.3389/fmicb.2015.00631

**Published:** 2015-06-24

**Authors:** Zhenfeng Liu, Amy E. Koid, Ramon Terrado, Victoria Campbell, David A. Caron, Karla B. Heidelberg

**Affiliations:** Department of Biological Sciences, University of Southern CaliforniaLos Angeles, CA, USA

**Keywords:** *Prymnesium parvum*, nutrient limitation, gene expression, polyketide synthase, algal bloom

## Abstract

*Prymnesium parvum* is a globally distributed prymnesiophyte alga commonly found in brackish water marine ecosystems and lakes. It possesses a suite of toxins with ichthyotoxic, cytotoxic and hemolytic effects which, along with its mixotrophic nutritional capabilities, allows it to form massive Ecosystem Disruptive Algal Blooms (EDABs). While blooms of high abundance coincide with high levels of nitrogen (N) and phosphorus (P), reports of field and laboratory studies have noted that *P. parvum* toxicity appears to be augmented at high N:P ratios or P-limiting conditions. Here we present the results of a comparative analysis of *P. parvum* RNA-Seq transcriptomes under nutrient replete conditions, and N or P deficiency to understand how this organism responds at the transcriptional level to varying nutrient conditions. In nutrient limited conditions we found diverse transcriptional responses for genes involved in nutrient uptake, protein synthesis and degradation, photosynthesis, and toxin production. As anticipated, when either N or P was limiting, transcription levels of genes encoding transporters for the respective nutrient were higher than those under replete condition. Ribosomal and lysosomal protein genes were expressed at higher levels under either nutrient-limited condition compared to the replete condition. Photosynthesis genes and polyketide synthase genes were more highly expressed under P-limitation but not under N-limitation. These results highlight the ability of *P. parvum* to mount a coordinated and varied cellular and physiological response to nutrient limitation. Results also provide potential marker genes for further evaluating the physiological response and toxin production of *P. parvum* populations during bloom formation or to changing environmental conditions.

## Introduction

*Prymnesium parvum* is a unicellular prymnesiophyte (haptophyte) alga that is found predominantly in brackish waters and lakes, as well as in some coastal marine ecosystems (Moestrup, 1994; Edvardsen and Paasche, 1998). Like all microbial eukaryotes, *P. parvum* requires nitrogen (N) and phosphorus (P) for basic physiological processes, but it is able to grow and bloom in a wide range of N and P concentrations. *P. parvum* is considered a model IIA mixotroph (Stoecker, 1998; Sanders, 2011), an alga that is primarily photosynthetic but one that can also use phagotrophy to supply essential substances needed for growth. Mixotrophic protists of this type are widespread in both freshwater and marine systems and can account for up to 50% of the bacterivory in some systems and also contribute to predation on other microbial taxa (Stoecker, 1998; Burkholder et al., 2008; Mitra and Flynn, 2010). The ability to ingest other organisms or take up dissolved organic matter is hypothesized to provide a competitive advantage for mixotrophic algae such as *P. parvum* when essential nutrients such as N and P, as well as iron, vitamins and other trace elements are limiting in the environment.

*P. parvum* can produce an array of toxins with hemolytic, cytotoxic, ichthyotoxic and possibly neurotoxic activities, at least some of which function in the acquisition of prey (Yariv and Hestrin, 1961; Shilo, 1971, 1981; Tillmann, 2003; Cichewicz and Hambright, 2010; Manning and La Claire, 2010). However, toxins produced by the alga likely constitute a suite of compounds with diverse cellular origins and biological activities (Manning and La Claire, 2010), of which only two have been isolated; prymnesin-1 and prymnesin-2 (Igarashi et al., 1999). The configuration of these cyclic polyethers have led researchers to postulate that, like the dinoflagellate toxins brevitoxin and okadaic acid, the synthesis of these molecules might involve polyketide synthase genes (Manning and La Claire, 2010; Freitag et al., 2011; Manning and La Claire, 2013).

Recurrent Ecosystem Disruptive Algal Blooms (EDABs) formed by *P. parvum* have resulted in significant economic losses in the aquaculture industry (Moestrup, 1994; Edvardsen and Paasche, 1998; Sunda et al., 2006), and these events appear to be increasing in frequency and geographical range. In recent years, *P. parvum* has spread throughout the northeast coast of the United States and into southwestern states, most notably Texas and Oklahoma (Aguiar and Kugrens, 2000; Watson, 2001; Hargraves and Maranda, 2002; Baker et al., 2009; Hambright et al., 2010). Blooms of *P. parvum* sufficient to result in fish kills tend to occur in environments characterized by high levels of macronutrients (Lindholm et al., 1999; Graneli and Salomon, 2010). Laboratory studies have indicated that *P. parvum* may produce toxins constitutively, but also that toxicity appears to increase under conditions of N- or P-limitation (Graneli and Johansson, 2003; Uronen et al., 2005; Graneli and Salomon, 2010). In accordance with those lab findings, toxicity in natural environments has been observed to be highest under high N:P ratios (Kaartvedt et al., 1991; Aure and Rey, 1992; Lindholm et al., 1999). These observations imply that highly toxic blooms of *P. parvum* capable of producing fish kills might occur where overall nutrient availability is sufficient to allow the alga to bloom but then subsequent conditions result in growth limitation that results in increased toxin production per cell.

Transcriptomic analysis provides an opportunity to probe the molecular underpinnings of the function and ecology of protists whose large genomes currently present financial and bioinformatics challenges for sequencing and assembly (McGettigan, 2013). Changes in gene expression of ecologically important species have been studied using methods such as expressed sequence tag (EST), microarray libraries or tag-based sequencing such as long-serial analysis of gene expression (long-SAGE) (von Dassow et al., 2009; Moustafa et al., 2010; Park et al., 2010; Wurch et al., 2011). Recently, higher throughput RNA-seq has become a common technology for gene expression analyses. These approaches allow for comparative analysis of differential gene expression, which can provide information into the physiological or cellular processes of protists whose large and complex genomes have not yet been sequenced.

Such studies have begun to provide insight into the transcriptional response of *P. parvum* to environmental conditions thought to affect its activities in natural communities. For example, one early study of a *P. parvum* EST library revealed the presence of phosphate transporter genes and genes that might be involved in polyketide synthesis (La Claire, 2006). A subsequent study using a combination of EST libraries and microarrays described differential expression of genes under N- and P- limitation, showing a strong response in P-limited cells by upregulating genes involved in the uptake of P (Beszteri et al., 2012). More recently, an RNA-Seq study focused on the mixotrophic nutrition of this alga, showing that different genes and pathways were upregulated depending on whether *P. parvum* was grown in the presence of bacteria or ciliates as prey (Liu et al., 2015). At a somewhat broader phylogenetic scale, comparative RNA-Seq transcriptome analysis described the differences between functional gene categories of four species of prymnesiophyte algae including *P. parvum*, and examined gene expression among phototrophic, heterotrophic and mixotrophic species of protists (Koid et al., 2014).

The present study investigated and compared specific changes in *P. parvum* gene expression under N- and P-limitation using RNA-Seq. Although this topic has been previously visited by Beszteri et al. (2012), advances in RNA-Seq technology used in this study offers improvement in both coverage of the transcriptome and resolution of gene expression levels. We explored how nutrient limitation impacts gene expression with an emphasis on the genes responding uniquely to N and P limitation relative to the alga growing in nutrient replete medium. Our results indicate that the expression levels of transporters for N and P were higher when the respective nutrient was limiting and that ribosomal and lysosomal proteins were more highly expressed when either N or P was limiting. In addition, photosynthesis genes were more highly expressed under P-limitation but not under N-limitation. Polyketide synthase genes were also more highly expressed under P-limitation, and may be related to the acquisition of extracellular P.

## Methods

### Culture conditions and RNA isolation

*Prymnesium parvum* UOBS-LP0109 (clone Texoma1) was isolated from Lake Texoma, Oklahoma, USA, by K. David Hambright, University of Oklahoma, transferred to the University of Southern California and made clonal and axenic by micropipetting single cells through rinses of sterile medium. The nutrient replete culture was grown in 2 L volumes in 2.8L ml Pyrex glass Fernbach flasks under specified conditions (Table 1). Nutrient-limited cultures were obtained by acclimation from the axenic replete culture and were prepared by at least three, 10–20% sequential inoculations at approximately 2-week intervals from the axenic replete culture.

**Table 1:** **Culture conditions for *Prymnesium parvum***.

**Culture**	**Media**	**Nutrients**	**Temp**	**L:D cycle**	**Irradiance^¶^**
1	L1^†^, -silica, 18ppt^*^	882 μM NO_3_^−^ 36.2 μM PO_4_^−3^	18°C	12:12	300 μE m^−2^ s^−1^
2	L1, -silica, N/100,18ppt	8.82 μM NO_3_^−^	18°C	12:12	300 μE m^−2^ s^−1^
3	L1, -silica, P/100, 18ppt	0.362 μM PO_4_^−3^	18°C	12:12	300 μE m^−2^ s^−1^

† *Guillard and Hargraves (1993) or https://ncma.bigelow.org*.

* *Salinity is indicated as parts per thousand (ppt)*.

¶ *Illumination was provided by Philips F20T12CW bulbs and measured using a QSL-100 sensor with QSP-170 deckbox (Biospherical Instruments, Inc.)*.

The replete treatment was harvested during mid-exponential growth phase, while the nutrient-limited treatments were harvested during stationary phase. RNA was isolated from the *P. parvum* cultures as described previously (Koid et al., 2014). Briefly, cultures were spun down, and the supernatant was decanted. The pellet was dissolved in TRI reagent (Ambion), and RNA was extracted from the homogenates using the Ribopure kit (Ambion). The eluted RNA was treated with DNase (Sigma) and cleaned and concentrated. The RNA was quantified using a Qubit 2.0 Fluorometer (Invitrogen) and run on an E-gel iBase with E-gel Gel EX 1% (Invitrogen) to check for nucleic acid quality.

### Library preparation and sequencing

RNA quality was assessed using the Agilent 2100 Bioanalyzer. Libraries were made using a previously published protocol (Koid et al., 2014). Briefly, Illumina's TruSeq RNA Sample Preparation Kit was used with 2 μg of RNA. The average insert size of each library ranged from 250 to 350 bp. Libraries were sequenced on an Illumina HiSeq 2000 to obtain 2 × 50 bp (paired-end) reads. Over 2 Gbp of sequence was generated per library. Library preparation and sequencing were performed as part of the Marine Microbial Eukaryote Transcriptome Sequencing Project (MMETSP) supported by the Gordon and Betty Moore Foundation (Keeling et al., 2014). The original sequence data are publicly available from NCBI Sequence Read Archive under accession number SRA166613 and sample IDs MMETSP0006_2, MMETSP0007, and MMETSP0814, for axenic, P-limited and N-limited treatment, respectively.

### Transcriptome assembly

Bioinformatic analysis procedures were adapted and developed from guidelines established by the MMETSP (Keeling et al., 2014). Sequences of all treatments were first checked for quality using the FASTX toolkit (http://hannonlab.cshl.edu/fastx_toolkit/index.html) with options “-p 80 -q 20” (at least 80% positions with quality score of at least 20). All remaining sequences were combined and assembled *de novo* using a combination of a de Brujin graph and overlap-based algorithms. Sequences were first assembled using ABySS v. 1.3.2 (Simpson et al., 2009) at four different k-mer settings of 19, 25, 31, and 37. The resulting four assemblies were merged using Trans-ABySS v. 1.4.4 (Robertson et al., 2010). Redundant contigs were removed using CD-Hit-EST v. 4.5.7 (Li and Godzik, 2006). The remaining contigs were then further assembled using CAP3 (Huang and Madan, 1999) with options “-p 99 -o 50 -k 0” (at least 50 bp overlap with 99% identity and no end clipping). Scaffolding of the resulting contigs was inferred using ABySS (Simpson et al., 2009). The GapCloser v. 1.12 application of the SOAPdenovo (Luo et al., 2012) was employed to fill in gaps in the assembly. Scaffolds were broken into contigs where gaps remained unfilled. Contigs shorter than 150 bp were discarded. CD-Hit-EST was used again to remove redundant contigs. Lastly, the contigs were searched against SILVA database (Quast et al., 2013) using BLAST (Altschul et al., 1990) to identify and remove rRNA sequences. Assemblies are available in the Supplementary Information or from the corresponding author.

### Transcriptome annotation

Protein-coding genes longer than 150 bp were predicted from the assembled transcriptome using ESTscan (Iseli et al., 1999). Genes were annotated at the 1e-5 level based on a variety of database searches including HMMER3 v. 3.1b1 (Zhang and Wood, 2003) searches against Pfam (Finn et al., 2010) and Tigrfam (Haft et al., 2001) databases and BLAST search against NCBI nr database. KEGG orthology and Gene Ontology terms were obtained using the KEGG annotation server (Moriya et al., 2007) and BLAST2GO (Conesa et al., 2005). Genes were annotated based on their homology to Pfam or Tigrfam protein families, KEGG or GO annotations, or best hit in nr database, in that order. Finally, automated annotation of selected genes in our datasets were manually inspected and curated.

### Differentially expressed genes

Sequences of all three datasets were aligned to the annotated genes of the assembled transcriptome using BWA (Li and Durbin, 2009). Read pairs that aligned to genes correctly were counted. Only read pairs mapped uniquely to genes were considered. Normalization and statistical analyses of the read counts of each gene were carried out in edgeR (Robinson et al., 2010). Pairwise comparisons between all three pairs of treatments were carried out using the “exact test” function in edgeR with dispersion set at 0.1. *P*-values were adjusted to false discovery rate using p.adjust in R (Benjamini and Hochberg, 1995). Only genes with adjusted *p* < 0.05 were accepted as having significantly different expression levels between different treatments. Read counts of 0 were replaced by 1 when calculating FPKM (fragments per kb per million fragments mapped) to avoid division by zero when calculating fold changes of gene expression between treatments. Genes whose FPKM values were below 1 in all treatments were ignored.

### Polyketide synthase analysis

Putative polyketide synthase (PKS) genes were identified by the automated annotation pipeline, and a local BLAST search against *Emiliania huxleyi* polyketide synthase sequences obtained from GenBank. The NRPS-PKS tool (Bachmann and Ravel, 2009) was used to identify the PKS domains present.

### Caveats and limitations of the study

We realize that transcriptome data in this study lacked replication. Therefore, we were purposely careful when interpreting the data. For example, inferences were made based on the expression patterns of a group of genes involved in the same function, never based on the expression pattern of a single gene. N-limited and P-limited treatments in this study were obtained by growing batch cultures of *P. parvum* to the stationary growth phase. Therefore, gene expression of these nutrient-limited cultures may have included responses that were a consequence of generalized stress, or changes related to life cycle processes associated with entering the stationary growth phase. However, gene expression observed for N- and P-limited treatments indicated several different responses to these nutrients, implying that transcriptomic responses of the alga were not overwhelmingly related to generalized stress or life cycle events associated with the stationary growth phase (which would presumably have been similar for the two nutrient-limited treatments).

## Results

### General transcriptome characteristics

Individual transcriptome characteristics are shown in Table 2. The assembled *P. parvum* transcriptome of the combined replete, N-limited and P-limited treatments contained 51,580 contigs and 42,862 genes for a total assembled transcriptome size of 43.95 Mbp. The number of putative protein-coding genes was comparable to other transcriptomes in the Gordon and Betty Moore Foundation MMETSP database and to the number of predicted genes in *Emiliania huxleyi*, another prymnesiophyte alga (Read et al., 2013). All necessary genes involved in glycolysis/gluconeogenesis, the TCA cycle, biosynthesis of amino acids, and nitrogen metabolism were present in the assembled transcriptome, indicating adequate sequencing depth to cover most actively transcribed genes.

**Table 2:** **Summary of *P. parvum* transcriptomes and assembly**.

	**Replete**	**P-limited**	**N-limited**
No. of read pairsLink: ^*^	19,277,859	18,785,652	11,404,203
Assembly statistics	51,580 contigs; 43.95 Mbp; N50 = 1276 bp; 42,862 predicted genes, 32.97 Mbp		
Reads mapped back to assembly	76.7%	55.5%	76.9%
Reads mapped back to genes	48.4%	22.8%	49.1%

* *After quality filtering*.

### Differentially expressed genes

**Figure 1:**
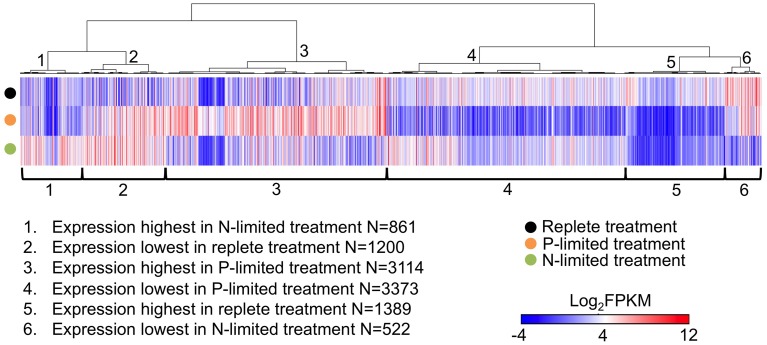
**Heatmap of expression levels (in the form of FPKM values) of all differentially expressed genes (between at least one pair of treatments) in three treatments**. Each thin column represents a single gene and its expression level in three treatments, Genes were arranged by hierarchical clustering (1-Pearson correlation, average linkage) of their expression patterns. When calculating Pearson correlation values, FPKM values smaller than zero were transformed to zero to reduce the impact of sub-zero FPKM values because they were mostly derived from very small read counts which have virtually no statistical significance. FPKM is the fragments per kilobase per million fragments mapped.

Approximately 25% (10,459) of the genes in our assembled transcriptome were differentially expressed in at least one pair of treatments. When clustered by their transcription patterns across three treatments, six different clusters of genes emerged. P-limitation seemed to have the largest impact on the transcriptome in terms of the numbers of differentially expressed genes. 3114 genes (cluster 3 in Figure 1) and 3373 genes (cluster 4) had highest and lowest expression levels in P-limited treatment, respectively. On the other hand, only 861 genes (cluster 1) and 522 genes (cluster 6) had highest and lowest expression levels in N-limited treatment. 1200 genes (cluster 2) were more highly expressed in both nutrient limited treatments, and 1389 genes (cluster 5) were most highly expressed in the replete treatment.

### Nitrogen uptake, transport, and assimilation

**Figure 2:**
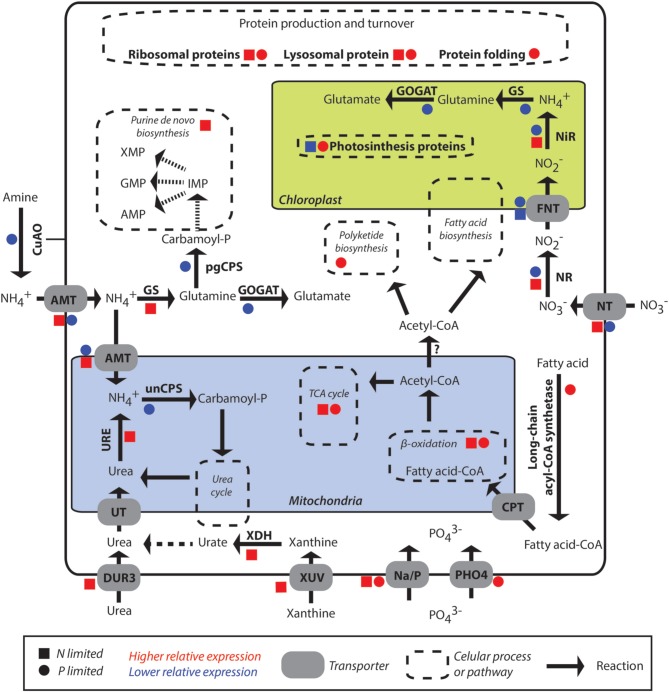
**Overview of differentially expressed pathways and genes of** ***P. parvum*** **grown to N- or P-limitation, relative to nutrient replete cultures**. Squares indicate response under N-limitation compared to the replete condition, while circles indicate response under P-limitation compared to the replete condition. Red and blue indicate up- and down-regulation respectively. AMT, ammonium transporter; CPT, carnithine palmitoyl transferase; CuAO, Copper amine oxidase; DUR3 and UT, urea transporter; FNT, formate/nitrite transporter; GOGAT, glutamate synthase; GS, glutamine synthetase; Na/P, sodium-dependent inorganic phosphate transporter; NiR, nitrite reductase; NR, nitrate reductase; NT, nitrate transporter; pgCPS, carbamoyl phosphate synthase (involved in pyrimidine syntheses, uses glutamine as substrate); PHO4, phosphate transporter of the pho4 family; unCPS, carbamoyl phosphate synthase (involved in the urea cycle, uses ammonium as substrate); URE, urease; XDH, xanthine dehydrogenase; XUV, xanthine-uracil permease.

**Figure 3:**
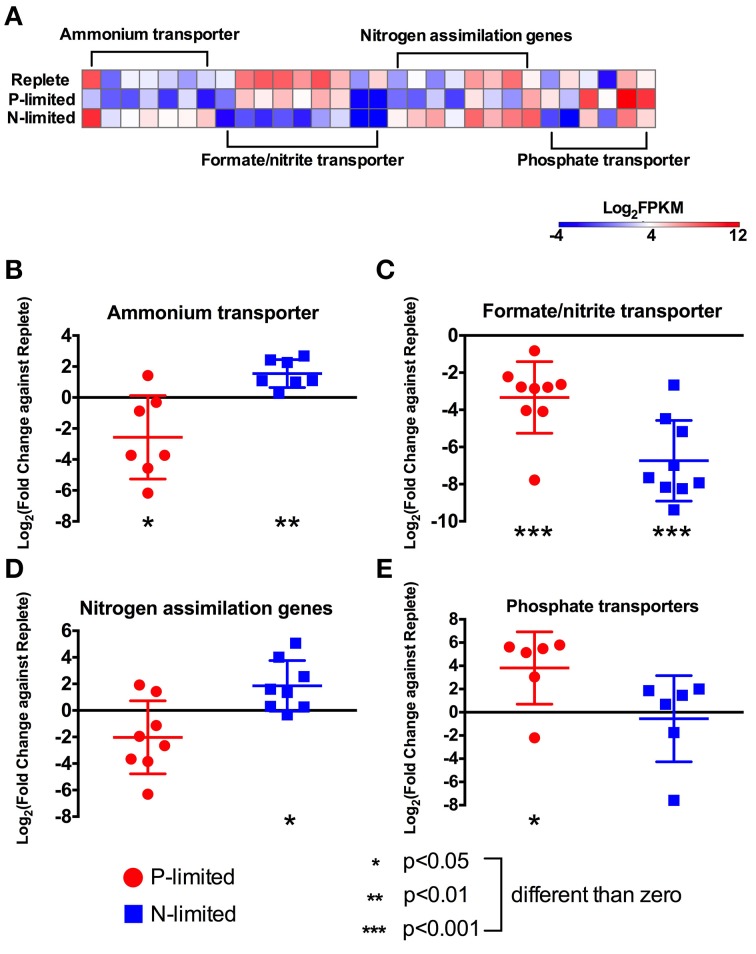
**Expression levels and fold changes of nitrogen and phosphorus metabolism genes. (A)** Heatmap of all differentially expressed genes involved in nitrogen and phosphorus metabolism. Log_2_ (fold change against replete treatment) in both P-limited and N-limited treatments were plotted for genes encoding ammonium transporters **(B)**, formate/nitrite transporters **(C)**, nitrogen assimilation genes **(D)**, and phosphate transporters **(E)**. For each category, a dot represents a single gene; the bar represents mean ± SD of the Log_2_ fold changes. For each category and each treatment, *t*-tests were used to test whether the Log_2_ fold change values were significantly different from 0, i.e., whether these genes were differentially expressed compared to the replete treatment, collectively. Different *p*-values are represented by different numbers of stars. For a list of these genes and their read counts and FPKM values, see Table S1.

Under N-limitation, changes in relative expression levels were observed for many genes related to N-uptake, transport and assimilation (Figure 2). Genes coding for proteins involved in nitrogen uptake, including nitrate reductase (NR), nitrite reductase (NiR), glutamine synthetase (GS) and glutamate synthase (GOGAT), had overall higher expression levels (*p* < 0.05) in the N-limited condition compared to the replete (Figures 3A,D). There were three isoforms of GS in our dataset. Two were annotated as glutamine synthetase III (GSIII) and most likely cytosolic isoforms. The third presented a signal peptide indicating a chloroplastidic localization, thus most likely being a putative glutamine synthetase II (GSII). One of the GSIII genes showed a marked response under N-limitation, being expressed at much higher level than that in the replete treatment. The other two GS genes and two GOGAT genes were not differentially expressed between the N-limited treatment and the replete treatment. Overall, expression levels of the nitrogen assimilation genes under P-limitation were not significantly different than those in the replete treatment (Figure 3D).

In addition to nitrogen metabolism genes, we found 20 putative inorganic nitrogen transporter genes: eight ammonium transporters, one nitrate transporter, and genes annotated as formate/nitrite transporters. The ammonium transporters and the nitrate transporter had higher expression levels (*p* < 0.01, *t*-test) under N-limitation compared to the replete condition but lower relative expression levels in the P-limited condition (*p* < 0.05, Figures 3A,B). In contrast, genes annotated as formate/nitrite transporters did not show the same pattern of regulation and presented a lower expression level in both N- and P-limited compared to the replete treatment (*p* < 0.001, Figure 3C). Two single copy urea transporters were detected in the transcriptome. The first was a urea permease of the UT family that appeared to be targeted to mitochondria and was not expressed differently in any treatment. The second was an active urea transporter (DUR3) with a higher relative expression level in the N-limited treatment compared to the replete treatment (Figure 3A, Table S1).

### Other nitrogen metabolism genes

Three different transporters for purines were expressed, possibly with affinities for different substrates (Supplemental Figure 1). Two of those were not differentially expressed among treatments, but one of the most highly expressed genes in the N-limited treatment corresponded to one of the purine transporters, a xanthine uracil permease (XUV); the expression level of this gene was higher in the N-limited treatment relative to the replete treatment (Table 3). A xanthine dehydrogenase that catalyzes the conversion of xanthine to urate (or uric acid) also showed a higher relative expression level in the N-limited treatment compared to the replete (Table 3).

**Table 3:** **Expression levels (in FPKM values) and read counts (in numbers of read uniquely aligned read pairs) of additional nitrogen metabolism genes not plotted in Figure 3**.

**ID**	**Annotation**	**FPKM (read count)**		
		**Replete**	**P-limited**	**N-limited**
63323	Putative urea transporter	57.3 (1159)	13.0 (115)	213.2 (2589)
135159_1	Putative nitrate transporter	877.6 (3185)	53.6 (85)	2527 (5505)
19338_2	Carbamoyl-phosphate synthase, large subunit	10.0 (330)	0.1 (2)	10.0 (198)
8693_1	Carbamoyl-phosphate synthase, large subunit	16.1 (731)	0.3 (6)	14.6 (400)
14637	Carbamoyl-phosphate synthase, large subunit	18.9 (945)	1.6 (36)	12.1 (362)
19322	Carbamoyl-phosphate synthase, large subunit	45.5 (1124)	8.6 (93)	40.9 (607)
99020	Carbamoyl-phosphate synthase, large subunit	2.3 (81)	0.1 (0)	2.3 (48)
19338_2	Carbamoyl-phosphate synthase, large subunit	10.0 (330)	0.1 (2)	10.0 (198)
57610	Xanthine dehydrogenase, molybdopterin binding subunit	13.2 (391)	5.7 (74)	50.3 (896)
18242	Uracil-xanthine permease	98.0 (4083)	25.1 (458)	948 (23705)
55569	Copper amine oxidase	6.5 (54)	0.6 (2)	6.8 (34)
62477_1	Urease, alpha subunit	10.9 (285)	9.7 (111)	41.5 (650)
92575	Argininosuccinate synthase	39.7 (589)	27.3 (177)	321.9 (2870)
63924	Argininosuccinate lyase	40.1 (679)	10.0 (74)	85.9 (873)

*Read counts of zero were converted to one when calculating FPKM values to avoid division by zero problems when the FPKM values were used to calculate fold changes between treatments*.

Genes related to the biosynthesis of purines also had higher relative expression levels in the N-limited treatment compared to the replete treatment, including the whole pathway converting ribose-5-phosphate to inosine monophosphate (IMP) and then interconverting IMP to xanthine monophosphate (XMP), guanine monophosphate (GMP), and adenosine monophosphate (AMP). Four of these genes, *purL*, *purM*, *purB*, and *purH*, were expressed at levels higher in the N-limited treatment compared to the replete treatment.

We detected a transcript coding for a copper amine oxidase (CuAO) with expression levels lower under P-limitation compared to the replete treatment (Table 3). The translated protein presents an α-helix at the N-terminal, suggesting that it corresponds to a membrane-bound CuAO (Figure 2). This gene was not differentially expressed under N-limitation.

There were also differences in expression levels of genes involved in intracellular nitrogen processing. Two types of carbamoyl phosphate synthase (CPS) genes were present in the combined transcriptome, previously designated in diatoms as unCPS and pgCPS (Allen et al., 2011). The former is localized to the mitochondria and is involved in the urea cycle, using ammonium as a substrate, while the latter is likely cytosolic and uses glutamine as a substrate in the first committed step of pyrimidine synthesis (Allen et al., 2011). There were six copies of pgCPS in the transcriptome, three of which were not differentially expressed in any of the treatments. The other three were not differentially expressed under N-limitation but had relatively lower expression under P-limitation (Figure 3A). Both unCPS genes found in the transcriptomes had lower transcription levels in the P-limited treatment, but neither were differentially expressed under N-limitation.

Two other genes involved in the urea cycle, argininosuccinate synthase and argininosuccinate lyase, also had higher expression levels under N-limitation (Table 3). Under P-limitation, argininosuccinate synthase was not differentially expressed while argininosuccinate lyase had a slightly lower expression level compared to the replete treatment (Table 3). The other urea cycle genes, arginine, and ornithine transcarbamoyltransferase were not differentially expressed among the three treatments.

### Phosphate transporters

Overall, expression levels of phosphate transporters were higher in the P-limited treatment, but were not statistically different in the N-limited treatment, compared to the replete treatment (Figure 3E). The most highly expressed gene in the P-limited treatment was one of two genes that were annotated as putative *pho4* family genes, a high-affinity sodium-phosphate transporter family that is generally regulated by phosphorous starvation in fungi (Dick et al., 2014) (Supplemental Figure 2). The expression level of this gene was higher in the P-limited treatment compared to the replete treatment. The second *pho4* gene was not differentially expressed. Three other putative sodium-dependent inorganic phosphate transporters most related to the Solute Carrier family (SLC) were more highly expressed in the P-limited condition, while another one annotated as a sodium-dependent inorganic phosphate transporter of the Major Facilitator Superfamily (MFS) was not differentially expressed.

### Photosynthesis proteins

Photosynthesis-related proteins found in the *P. parvum* transcriptome consisted of chlorophyll A-B binding proteins and light-harvesting proteins. Most photosynthesis-related genes are encoded in the chloroplast genome, and thus were not recovered in our datasets due to the poly-A+ enrichment technique used.

**Figure 4:**
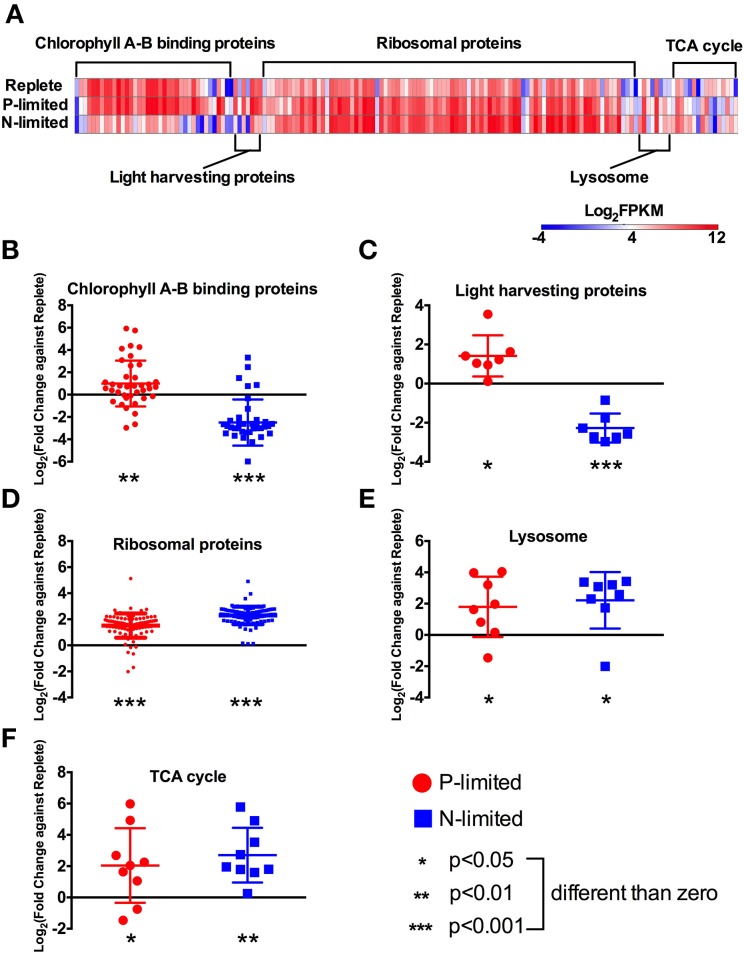
**Expression levels and fold changes of selected functions and pathways. (A)** Heatmap of all differentially expressed genes involved in targeted functions/pathways. Log_2_ (fold change against the replete treatment) in both P-limited and N-limited treatments were plotted for genes encoding chlorophyll A-B binding proteins **(B)**, light harvesting proteins **(C)**, ribosomal proteins **(D)**, lysosomal proteins **(E)**, and TCA cycle proteins **(F)**. For each category, a dot represents a single gene; bar represent mean ± SD of the Log_2_ fold change. For each category and each treatment, *t*-tests were carried out to test whether the Log_2_ fold change values were significantly different from 0, i.e., whether these genes are differentially expressed compared to the replete treatment, collectively. Different *p*-values are represented by different numbers of stars. For a list of these genes and their read counts and FPKM values, see Table S1.

We found 38 chlorophyll A-B binding protein genes that were differentially expressed among the treatments. Most of these genes had lowest expression levels in the N-limited treatment (Figure 4A). Overall, these genes had higher expression levels under P-limitation (*p* < 0.01), and lower expression levels under N-limitation (*p* < 0.001), compared to the replete treatment (Figure 4B).

Relative expression levels of genes coding for light-harvesting proteins also exhibited opposite patterns under P- and N-limitation. That is, transcription levels of these genes were generally higher in the P-limited treatment (*p* < 0.05), but were lower in the N-limited treatment relative to the replete treatment (*p* < 0.001, Figure 4C).

### Protein synthesis and degradation

The genes involved in protein synthesis in our dataset were ribosomal proteins that are involved in the creation of new proteins and lysosomal proteolytic enzymes. Ninety different genes encoding ribosomal proteins are differentially expressed among treatments. Overall, they had higher expression levels in both P-limited and N-limited treatments compared to the replete treatment (*p* < 0.001, Figure 4D).

A group of proteolytic enzymes that were annotated by KEGG as lysosomal proteins also had higher expression levels under both P- and N-limitation (*p* < 0.05, Figure 4E). These genes code for 3 aspartyl proteases, 3 cysteine proteases, and 2 serine carboxypeptidases. Six of these eight genes were expressed at higher levels under P-limitation, while seven were expressed at higher levels under N-limitation.

### Polyketide synthase genes

There were 15 differentially expressed polyketide synthase (PKS) genes in our dataset. Nine genes had higher expression levels in the P-limited treatment. Among them, transcripts of 5 genes were only detected in the P-limited treatment, while an additional 4 PKS genes were expressed at higher levels under P-limitation compared to the replete treatment. Under N-limitation, 3 genes had higher and 7 genes had lower expression levels compared to the replete condition.

The PKS domains found in the 15 genes included the ketosynthase (KS), ketoreductase (KR), dehydratase (DH), and acyl-carrier-protein (ACP) domains. Of these domains, the KS, KR and ACP domains are the minimum required domains for PKS synthesis. The gene with the most number of domains, containing a KR domain followed by two ACP domains, had higher expression levels in both nutrient limited treatments. The two genes with KS domains were more highly expressed in the replete treatment compared to both the N-limited and P-limited treatments. One gene with an ACP domain and another with two ACP domains were also more highly expressed in the replete treatment relative to the nutrient-limited treatments. The other genes annotated as PKS genes did not have domain annotations.

### Fatty acid oxidation and tricarboxylic acid (TCA) cycle genes

Many genes in the fatty acid oxidation pathway were expressed at higher levels under both P- and N-limitation compared to replete condition (Figure 2). Among them were five copies of long-chain acyl-CoA synthetase, two acyl CoA dehydrogenases, and two encoding enoyl-CoA hydratase. Many of the genes involved in the TCA cycle were also similarly expressed at higher levels under both nutrient-limited treatments compared to replete (Figures 2, 4F). They included pyruvate carboxylase, two putative isocitrate dehydrogenase genes, aconitate hydratase fumerase, malate dehydrogenase, 2-oxoglutarate dehydrogenase and succinyl-coA synthetase (Table S1). However, citrate synthase was not differentially expressed in any treatments.

## Discussion

### Nitrogen uptake and metabolism genes responded to N limitation

Nitrogen is essential for cell maintenance, growth and proliferation, and synthesis of proteins, DNA, RNA and photosynthetic pigments. Under N-limitation, *P. parvum* must meet nitrogen requirements either by increasing the expression of genes used to acquire exogenous nitrogen or by repartitioning intracellular pools of nitrogen. Many nitrogen transporters in the assembled transcriptome including those for ammonium, nitrate, nitrite and urea were more highly expressed under N-limitation compared to the nutrient-replete condition (Figures 2, 3). A similar response in nitrogen transporters of *P. parvum* under N-limitation was not observed in a previous study (Beszteri et al., 2012). Expression levels of most of these genes are not very high (in most cases <1% of the transcriptome, often lower than 0.1%), therefore the apparent discrepancy could be the result of inadequate sequencing depth of the previous study. However, a nitrate transporter and an ammonium transporter were upregulated in a related prymnesiophyte, *E. huxleyi*, and the pelagophyte, *A. anophagefferens*, under nitrogen stress (Dyhrman et al., 2006; Wurch et al., 2011).

General but non-universal preference among phytoplankton for ammonium uptake and assimilation over nitrate appeared to also apply to *P. parvum* (Dortch, 1990). Preference for ammonium is energetically favorable as it can be incorporated directly into organic matter via the glutamine-synthetase-glutamate synthase (GS-GOGAT) cycle, while nitrate requires energy for reduction to ammonium before it can be utilized in biological processes (Figure 2). Seven ammonium transporter genes in our assembled transcriptome were upregulated compared to just one nitrate transporter. Diatoms, which tend to readily assimilate nitrate (Sarthou et al., 2005), have also been shown to have more ammonium transporters than nitrate transporters, although the ratio is closer to 2:1 (Hildebrand, 2005).

In addition to increasing gene expression associated with the uptake of exogenous nitrogen, our results indicated an increase in transcription of nitrogen assimilation genes, specifically for the reduction of nitrate to ammonium and for genes in the GS-GOGAT cycle. In our study, the cytosolic GSIII genes had higher expression levels under N-limitation, while the chloroplastic GSII did not. The former converts ammonium that is taken up from outside the cell into glutamine while the latter uses the ammonium that is reduced from nitrate within the cell. This may imply that recycled reduced nitrogen from extracellular sources such as proteins might replace nitrate reduction as the main nitrogen source under N-limitation. Conversely, GSIII could be upregulated as a consequence of greater mobilization of intracellular N-pools accompanying degradation of proteins or urea, liberating ammonia that is then reincorporated into other cellular constituents.

### Other nitrogen metabolism genes

We also observed a higher relative expression for one purine transporter under N-limited conditions. These findings are similar to results reported for the prymnesiophyte, *E. huxleyi*, which was able to grow on purines (Palenik and Henson, 1997) using transporters induced by N-limitation (Shah and Syrett, 1984). Furthermore, at least two important enzymes in the catabolic process of purines, xanthine dehydrogenase and urease had higher relative expression levels under N-limited conditions. Together with a higher relative expression of the active urea transporter DUR3 (Figure 2), our results indicated that *P. parvum* grown under N-limiting conditions exhibited a coordinated response to scavenge N from organic substrates (Figure 2), contrasting with a previous report that did not observe this response (Beszteri et al., 2012). Similar to the observation by Beszteri et al. (2012), however, a transcript coding for a copper-amine oxidase had lower expression levels in the P-limited treatment compared to the replete condition (CuAO in Figure 2). CuAO has a α-helix at the N-terminal, suggesting that it might correspond to the same membrane-bound enzyme described in *P. parvum* that oxidizes primary amines to produce extracellular ammonium (Palenik and Morel, 1991). This activity combined with the uptake of ammonium suggests that this enzyme plays a role in cell nutrition. The lower relative expression of this enzyme is thus in line with the lower relative expression of ammonium transporters in the P-limited treatment.

### Phosphate transporters responded to P limitation

The most highly expressed gene under P-limitation was a phosphate transporter belonging to the *Pho4* superfamily of phosphate permeases. The *Pho4* superfamily includes both high- and low-affinity phosphate transporters. In yeast, the low-affinity phosphate transport system, thought to be constitutively expressed, satisfies the cellular requirement for inorganic phosphate when external levels of phosphate are high or sufficient. In contrast, the high-affinity phosphate transport system is used when ambient phosphate levels are limiting to growth (Persson et al., 2003). In other microbial eukaryotes, genes with homology to *pho4* have been found in species from different phylogenetic groups including chlorophytes, *Tetraselmis chui*, and *Dunialiella viridis* (Chung et al., 2003; Li et al., 2006), several prasinophytes (Monier et al., 2012), the pelagophyte *A. anophagefferens* (Wurch et al., 2011), and the prymnesiophyte *E. huxleyi* (Dyhrman et al., 2006). Experimental evidence and the phylogenetic placements of these *pho4* genes suggest that they are all high-affinity transporters, and that P-depletion induces upregulation of these genes (Wykoff et al., 2007).

In addition to the putative *pho4* gene, other transporters were detected in this study that were annotated as sodium-dependent inorganic phosphate transporters. These transporters couple the uptake of inorganic phosphorus to the ionic gradient formed by the movement of Na^+^ (Persson et al., 2003). While three of these transporters were upregulated under P-limitation, one was downregulated compared to the replete condition. We suggest that differential regulation of these genes in *P. parvum* with putatively similar function may be a result of their different affinities for transporting inorganic phosphorus. Differential upregulation in *P. parvum* of the two phosphate permeases and two acid phosphatases under P limitation was described previously (Beszteri et al., 2012). In this study, one of each type was upregulated indicating that there could be different phosphorus acquisition systems depending on the nutrient status of the cell. Taken together, the two different phosphate transporter families indicate different cellular responses to acquire more phosphorus when the nutrient is limiting to growth of *P. parvum*.

### Photosynthesis responded differently to N and P limitation

Genes associated with photosynthesis in *P. parvum* in our study responded differently to N and P limitations. Under P-limitation, higher expression levels of photosynthesis genes were observed relative to the N-limited treatment (Figures 4B,C). Expression levels of genes related to photosynthesis were higher under P-limitation possibly to compensate for a reduction in photosynthesis efficiency (F_v_/F_m_), which has been observed for some algae under phosphorus limitation (Shen and Song, 2007). Alternatively, we speculate that the relative upregulation of photosynthesis genes under phosphorus limitation may provide energy necessary to fuel increased cellular reorganization and/or toxin synthesis under P-limited conditions.

Maintenance of the photosynthetic apparatus is thought to require much higher levels of N than P because of the large amounts of N-rich chlorophylls. This may explain why N-limitation had a greater negative effect on the expression levels of photosynthesis genes than P-limitation, a situation observed for several other photosynthetic microorganisms. In the unicellular cyanobacterium *Synechococcus* spp., components of the cell's photosynthetic machinery such as chlorophyll, phycocyanin, and phycobilisomes responded differently to N- and P-deprivation, generally with a greater negative effect observed under nitrogen limitation (Collier and Grossman, 1992). Similarly, a decline in the number of photosystem II reaction centers in the green alga, *Chlamydomonas* sp. was found to be greater under N-deprivation compared to P-deprivation (Grossman, 2000). The coccolithophore, *E. huxleyi*, also yielded results similar to our study, wherein five putative fucoxanthin chlorophyll a-b binding protein genes were found to be highly upregulated under P-limitation and downregulated under N-limitation (Dyhrman et al., 2006). Genes involved in photosynthesis were also downregulated in *Chlamydomonas reinhardtii* under N-limitation (Miller et al., 2010).

### Genes involved in protein production and turnover responded to N- and P-limitation

Our results indicate that *P. parvum* increased the expression of genes involved in both protein synthesis and degradation under both N- and P- limitation (Figures 4D,E). Similarly, multiple rRNA transcripts were shown to be upregulated under P-limitation in *E. huxleyi* (Dyhrman et al., 2006), although this is not a universal transcriptional response, as observed for the diatom *T. pseudonana* (Dyhrman et al., 2012). The higher expression levels of ribosomal genes was somewhat surprising under N-limitation, as protein synthesis might be hampered due to large requirements of N. However, the higher expression levels of lysosome-related genes (Figure 4E) implies a higher protein degradation rate, which might provide the amino acids required for new protein synthesis. Our results suggest that there is an overall response for protein and amino acid recycling in *P. parvum* under nutrient limitation. Under these conditions, we speculate that large amounts of proteins might need to be recycled to make other proteins that directly address nutrient deficiencies, such as ammonium and phosphate transporters, or cellular reorganization associated with the cessation of population growth in stationary phase.

### Polyketide synthase (PKS) genes respond to nutrient limitation

PKS compounds have been implicated as the basis for toxins in many dinoflagellates (Kellmann et al., 2010) and are thought to be responsible for some if not all of the toxins present in prymnesiophytes (Freitag et al., 2011). Two *P. parvum* toxins have been isolated to date, prymnesin1 and prymnesin2 (Igarashi et al., 1999; Manning and La Claire, 2010) although other yet uncharacterized toxins may be present (Cichewicz and Hambright, 2010). Prymnesins are polyether compounds similar to other algal toxins such as brevitoxin and okadaic acid, which are produced by Type I PKS genes. PKS genes have been found in the transcriptome of *P. parvum* and other related prymnesiophytes, with the ketosyntase (KS) domain of the PKS genes clustered into two separate clades, one comprising prymnesiophyte-specific sequences and one apparently of diverse bacterial and protistan origin (Koid et al., 2014).

Our dataset contained many different PKS genes that exhibited different expression patterns. However, 9 of the 15 genes had higher expression levels under P-limitation, consistent with previous observations of increased toxicity under P-limitation (Graneli and Johansson, 2003; Uronen et al., 2005). It is not yet known which of the PKS genes participate in toxin production, and it is also possible that some PKS genes participate in the synthesis of different toxins (Manning and La Claire, 2010). However, the PKS genes that responded positively to nutrient limitation in the present study provide targets for further study focused on identifying genes and pathways involved in toxin production in *P. parvum*. Under P-limitation, increased toxin production and release may help *P. parvum* obtain more extracellular phosphorus from lysed cells of other organisms (Graneli and Salomon, 2010). The higher expression of some PKS genes in our study is consistent with the suggestion that P-limited cells have higher toxicity than nutrient-replete cells, which might be part of a coordinated physiological response to P-limitation. We hypothesize that the upregulation of photosynthetic genes under P limitation described above may provide cells with the energy necessary for toxin production.

### Fatty acid oxidation and the TCA cycle

Fatty acid breakdown involves multiple cycles of β-oxidation—depending on the length of the fatty acid—and results in acetyl-CoA and NADH and FADH_2_. We observed an increase in the expression levels of many genes involved in fatty acid (FA) oxidation in response to both types of nutrient limitation (Figure 2). This was an unexpected result based on observations that phytoplankton such as the green alga, *Chlamydomonas*, are thought not to synthesize fatty acids under nutrient limitation (Moseley et al., 2006). While many of these enzymes function bidirectionally, acyl-CoA dehydrogenase is unidirectional. Therefore, its higher expression level under P- and N-limitation might indicate that other enzymes are also catalyzing reactions that result in an increase in acetyl-CoA. Acetyl-CoA can feed into a number of different downstream pathways, one of which is the TCA cycle, while another is PKS synthesis (Dewick, 2002). The expression levels of PKS genes rose under P-limitation, as noted above. TCA cycle genes had higher expression levels under both P and N-limitation (Figure 4F), implying one possible use of the additional acetyl-CoA might be energy production through the TCA cycle. The increase in expression levels of TCA cycles could also have many other implications such as possible increased production of 2-oxoglutarate for the GS/GOGAT cycle.

## Conclusions

*P. parvum* exhibited two types of responses under N- and P-limitation: responses that were specific to either P or N limitation, and a general response common to both nutrient stress conditions. P-limitation resulted in the increased expression of phosphate transporters, photosynthesis genes and polyketide synthase genes. Toxin synthesis might be related to the acquisition of extracellular P when that nutrient is limiting, while the relative upregulation of photosynthesis genes might provide the energy necessary to fuel the increased cellular activity. N- and P-limitation both resulted in an increase in genes involved in protein synthesis and turnover, fatty acid oxidation and the TCA cycle. Under N limitation, cells upregulated genes associated with N assimilation including a myriad of different transporters and nitrogen-related pathways. This robust cellular response included the processing or reallocation of intracellular nitrogen, such as seen in the *de novo* purine biosynthesis pathway. Taken together, the results of this study highlight the ability of *P. parvum* to mount a coordinated and varied cellular and physiological response to nutrient limitation.

### Conflict of interest statement

The Associate Editor Senjie Lin declares that, despite having collaborated with author David A. Caron, the review process was handled objectively and no conflict of interest exists. The authors declare that the research was conducted in the absence of any commercial or financial relationships that could be construed as a potential conflict of interest.
