# Supplementary material for: Changes in gene expression of Prymnesium parvum induced by nitrogen and phosphorus limitation
Source: Front Microbiol. 2015 Jun 24;6:631. doi: 10.3389/fmicb.2015.00631 (PMC4478897; doi:10.3389/fmicb.2015.00631)
Supplement: Supplementary file 1 [file Table1.DOCX]

**Table S1**. Expression levels (in FPKM values) and read counts (in numbers of read uniquely aligned read pairs) of genes discussed in this study. Read counts of zero were converted to one when calculating FPKM values to avoid division by zero problems when the FPKM values were used to calculate fold changes between treatments.

| **ID** | **Annotation** | **FPKM(read count)** | | |
| --- | --- | --- | --- | --- |
|  |  | **Replete** | **P-limited** | **N-limited** |
| **Nitrogen metabolism** | | | | |
| 135276_2 | ammonium transporter | 450.0(6919) | 6.5(44) | 963.7(8894) |
| 65858 | ammonium transporter | 1.6(27) | 1.4(10) | 10.3(104) |
| 99615 | ammonium transporter | 13.6(29) | 1.1(0) | 16.4(21) |
| 897 | ammonium transporter | 12.8(244) | 7.3(61) | 25.6(293) |
| 65702 | ammonium transporter | 8.5(29) | 0.7(0) | 18.1(37) |
| 99491 | ammonium transporter | 3.6(66) | 10.1(81) | 19.4(213) |
| 9639 | ammonium transporter family protein | 9.2(155) | 0.4(3) | 44.1(445) |
| 63323 | putative urea transporter | 57.3(1159) | 13.0(115) | 213.2(2589) |
| 135159_1 | putative nitrate transporter | 877.6(3185) | 53.6(85) | 2527(5505) |
| 134706 | formate/nitrite transporter | 13.0(60) | 2.0(4) | 0.4(0) |
| 115477 | formate/nitrite transporter | 210.0(335) | 47.3(33) | 1.0(1) |
| 135478_1 | formate/nitrite transporter | 354.1(1110) | 22.6(31) | 0.5(1) |
| 135478_2 | formate/nitrite transporter | 341.6(1514) | 52.6(102) | 1.1(3) |
| 135478_3 | formate/nitrite transporter | 110.7(406) | 18.7(30) | 0.5(1) |
| 135478_4 | formate/nitrite transporter | 467.0(2536) | 67.8(161) | 3.7(12) |
| 2658 | formate/nitrite transporter | 58.8(550) | 34.9(143) | 9.3(52) |
| 8560 | formate/nitrite transporter | 3.2(37) | 0.2(1) | 0.1(0) |
| 8903 | formate/nitrite transporter | 37.2(478) | 0.2(0) | 0.1(0) |
| 14951 | nitrite reductase 4Fe-4S domain | 3.6(86) | 1.7(18) | 21.3(303) |
| 1636 | glutamine synthetase type III N terminal | 16.2(412) | 1.4(15) | 49.3(752) |
| 18602 | glutamine synthetase type III N terminal | 2.7(64) | 7.5(79) | 89.3(1287) |
| 98222 | glutamine synthetase, catalytic domain | 11.4(156) | 0.8(5) | 13.6(112) |
| 59328 | glutamate synthase, NADH/NADPH, small subunit | 104.7(1905) | 28.4(226) | 267.5(2921) |
| 61649 | putative glutamate synthase | 53.0(2577) | 8.8(188) | 65.5(1913) |
| 97211 | nitrate reductase | 245.8(7065) | 3.3(41) | 196.4(3388) |
| 19338_2 | carbamoyl-phosphate synthase, large subunit | 10.0(330) | 0.1(2) | 10.0(198) |
| 8693_1 | carbamoyl-phosphate synthase, large subunit | 16.1(731) | 0.3(6) | 14.6(400) |
| 14637 | carbamoyl-phosphate synthase, large subunit | 18.9(945) | 1.6(36) | 12.1(362) |
| 19322 | carbamoyl-phosphate synthase, large subunit | 45.5(1124) | 8.6(93) | 40.9(607) |
| 99020 | carbamoyl-phosphate synthase, large subunit | 2.3(81) | 0.1(0) | 2.3(48) |
| 19338_2 | carbamoyl-phosphate synthase, large subunit | 10.0(330) | 0.1(2) | 10.0(198) |
| **Phosphate transporters** | | | | |
| 13393 | Na(+)-dependent inorganic phosphate cotransporter | 3.0(28) | 26.3(106) | 0.9(5) |
| 44826 | Na(+)-dependent inorganic phosphate cotransporter | 29.3(319) | 6.7(32) | 0.2(1) |
| 17036 | sodium-dependent inorganic phosphate (Pi) transporter | 12.6(214) | 591.6(4400) | 45.7(467) |
| 65765 | [sodium-dependent inorganic phosphate (Pi) transporter | 0.4(5) | 19.0(113) | 1.5(12) |
| 135755_1 | phosphate transporter family protein | 72.7(904) | 4238(23053) | 199.6(1490) |
| 135755_2 | phosphate transporter family protein | 21.8(59) | 808.8(957) | 35.1(57) |
| **Photosynthesis related proteins** | | | | |
| 11500 | chlorophyll A-B binding protein | 5.3(17) | 0.7(0) | 0.5(0) |
| 43082 | chlorophyll A-B binding protein | 97.9(1002) | 16.3(73) | 7.7(47) |
| 9803 | chlorophyll A-B binding protein | 69.0(607) | 22.1(85) | 6.2(33) |
| 98977 | chlorophyll A-B binding protein | 652.5(3330) | 297.5(664) | 106.4(326) |
| 135153_2 | chlorophyll A-B binding protein | 1045(8814) | 587.5(2167) | 129.6(656) |
| 82918 | chlorophyll A-B binding protein | 1080(1603) | 644.3(418) | 102.2(91) |
| 9200 | chlorophyll A-B binding protein | 228.8(2468) | 156.3(737) | 20.5(133) |
| 135526_2 | chlorophyll A-B binding protein | 739.7(3720) | 618.0(1359) | 105.7(319) |
| 18545 | chlorophyll A-B binding protein | 698.0(8186) | 599.0(3072) | 85.4(601) |
| 97071 | chlorophyll A-B binding protein | 203.6(1569) | 193.5(652) | 25.1(116) |
| 135549_1 | chlorophyll A-B binding protein | 1617(6353) | 1598(2745) | 321.5(758) |
| 3978 | chlorophyll A-B binding protein | 413.7(4999) | 421.5(2227) | 21.8(158) |
| 135528 | chlorophyll A-B binding protein | 1006(8450) | 1232(4527) | 164.5(829) |
| 133638 | chlorophyll A-B binding protein | 385.3(1923) | 472.8(1032) | 80.8(242) |
| 15115 | chlorophyll A-B binding protein | 49.6(595) | 67.8(356) | 8.6(62) |
| 18068 | chlorophyll A-B binding protein | 166.6(1999) | 228.9(1201) | 40.3(290) |
| 14612 | chlorophyll A-B binding protein | 125.3(907) | 192.0(608) | 23.0(100) |
| 106720 | chlorophyll A-B binding protein | 968.7(1663) | 1510(1134) | 137.8(142) |
| 18164 | chlorophyll A-B binding protein | 1004(8445) | 1704(6265) | 100.7(508) |
| 98475 | chlorophyll A-B binding protein | 925.9(2747) | 1597(2072) | 115.1(205) |
| 135249_1 | chlorophyll A-B binding protein | 330.1(2088) | 599.4(1658) | 22.7(86) |
| 135683_1 | chlorophyll A-B binding protein | 1239(8844) | 2258(7049) | 155.0(664) |
| 10322 | chlorophyll A-B binding protein | 340.2(619) | 632.2(503) | 55.9(61) |
| 133679 | chlorophyll A-B binding protein | 332.9(1500) | 662.7(1306) | 45.5(123) |
| 135409_1 | chlorophyll A-B binding protein | 590.7(5511) | 1178(4809) | 101.4(568) |
| 135249_2 | chlorophyll A-B binding protein | 90.1(465) | 204.3(461) | 6.5(20) |
| 9696 | chlorophyll A-B binding protein | 96.5(1264) | 223.3(1279) | 1.5(12) |
| 96736 | chlorophyll A-B binding protein | 67.5(345) | 203.5(455) | 10.8(33) |
| 136029_2 | chlorophyll A-B binding protein | 496.0(4665) | 1584(6516) | 65.0(367) |
| 16802 | chlorophyll A-B binding protein | 83.4(782) | 530.2(2174) | 1.1(6) |
| 15098 | chlorophyll A-B binding protein | 28.0(254) | 189.9(753) | 0.4(2) |
| 52754 | chlorophyll A-B binding protein | 15.3(131) | 136.8(512) | 43.0(221) |
| 15389 | chlorophyll A-B binding protein | 3.2(23) | 36.9(117) | 2.5(11) |
| 9703 | chlorophyll A-B binding protein | 0.7(4) | 12.7(32) | 0.3(1) |
| 16414 | chlorophyll A-B binding protein | 141.4(388) | 2848(3417) | 257.5(424) |
| 95534 | chlorophyll A-B binding protein | 21.8(159) | 477.3(1521) | 37.3(163) |
| 17949 | chlorophyll A-B binding protein | 0.3(3) | 19.1(74) | 1.9(10) |
| 19304_1 | chlorophyll A-B binding protein | 0.2(0) | 10.2(28) | 1.6(6) |
| 136047_1 | chloroplast light harvesting protein | 241.4(1225) | 491.1(1090) | 30.9(94) |
| 136047_2 | chloroplast light harvesting protein | 4.7(67) | 15.1(95) | 0.7(6) |
| 66322 | chloroplast light harvesting protein | 204.9(862) | 443.5(816) | 60.6(153) |
| 131662_1 | chloroplast light harvesting protein | 3.2(11) | 39.3(59) | 0.5(1) |
| 17002 | chloroplast light harvesting protein | 516.1(2966) | 1270(3193) | 287.0(990) |
| 135526_1 | chloroplast light harvesting protein | 256.5(670) | 716.0(818) | 52.9(83) |
| 17413 | chloroplast light harvesting protein | 345.1(528) | 391.6(262) | 57.7(53) |
| **Ribosomal proteins** | | | | |
| 64478 | ribosomal protein S10p/S20e | 7.5(100) | 51.0(297) | 8.1(65) |
| 3710 | ribosomal protein S17 | 29.3(225) | 107.8(362) | 32.1(148) |
| 63846 | ribosomal protein L14p/L23e | 29.0(185) | 100.5(280) | 32.4(124) |
| 6710 | ribosomal L18p/L5e | 93.7(547) | 62.3(159) | 209.9(736) |
| 13104 | ribosomal protein L1 | 80.3(1078) | 26.1(153) | 190.1(1532) |
| 18392 | ribosomal protein L13 | 171.2(1597) | 123.0(502) | 432.5(2422) |
| 7919 | ribosomal protein S1 | 87.7(1119) | 22.8(127) | 234.3(1795) |
| 134933_1 | ribosomal protein S21e | 51.3(410) | 218.8(764) | 154.0(738) |
| 98978 | ribosomal protein S17 | 63.5(250) | 174.2(300) | 196.8(465) |
| 103117 | 60S ribosomal protein, putative | 217.8(313) | 857.8(539) | 699.2(603) |
| 134933_2 | ribosomal protein S21e | 50.5(481) | 223.7(932) | 162.7(930) |
| 131995 | ribosomal protein L44 | 86.2(296) | 422.3(634) | 286.8(591) |
| 123776 | ribosomal protein L35Ae | 342.9(563) | 1229.7(883) | 1145(1129) |
| 135378_2 | ribosomal protein L28e | 25.3(150) | 121.4(315) | 89.3(318) |
| 136103_1 | ribosomal protein S28e | 111.1(370) | 541.7(789) | 394.7(789) |
| 15185 | ribosomal protein L11 | 7.8(56) | 11.1(35) | 27.8(120) |
| 134982_2 | ribosomal protein L1p/L10e family | 334.7(3947) | 743.1(3832) | 1202(8514) |
| 1506 | ribosomal protein L19e | 267.1(1388) | 337.5(767) | 960.5(2996) |
| 136046_1 | ribosomal protein S27 | 187.5(782) | 1337(2440) | 682.3(1708) |
| 12805 | ribosomal protein S26e | 193.0(1210) | 744.0(2040) | 716.9(2698) |
| 135623_1 | ribosomal protein L37Ae | 190.3(1642) | 688.4(2598) | 713.7(3697) |
| 99433 | ribosomal protein L10 | 68.0(732) | 63.9(301) | 257.3(1663) |
| 16888 | ribosomal protein L19e | 454.1(805) | 855.3(663) | 1751(1864) |
| 135451_1 | ribosomal protein L22e | 81.7(398) | 233.8(498) | 315.7(923) |
| 16913 | ribosomal protein S17 | 453.4(753) | 1695(1231) | 1836(1831) |
| 134874_1 | ribosomal L40e family | 306.6(2111) | 1152(3469) | 1308(5407) |
| 135062_1 | ribosomal protein S15e | 366.3(2560) | 1168(3572) | 1578(6622) |
| 9547 | ribosomal protein L12e | 2.1(17) | 7.5(26) | 9.3(44) |
| 6514 | ribosomal protein L9e | 172.9(734) | 660.4(1226) | 749.2(1909) |
| 63443 | 60S ribosomal protein, putative | 26.9(97) | 152.6(241) | 116.7(253) |
| 134922_1 | ribosomal protein S5e | 107.8(1164) | 236.0(1114) | 469.3(3041) |
| 135009_2 | ribosomal protein L24e | 155.4(925) | 893.6(2326) | 685.5(2449) |
| 18898 | ribosomal protein L31e | 200.7(1614) | 572.7(2014) | 889.7(4295) |
| 135510_2 | ribosomal protein L14 | 45.6(191) | 199.2(365) | 202.8(510) |
| 98612 | ribosomal protein L37e | 23.2(131) | 117.9(291) | 107.1(363) |
| 19194 | ribosomal protein S13e | 116.8(726) | 302.5(822) | 540.5(2016) |
| 135510_1 | ribosomal protein L14 | 145.3(983) | 404.3(1196) | 679.3(2758) |
| 135613_2 | ribosomal protein S16e | 142.7(603) | 544.8(1007) | 667.4(1693) |
| 135378_1 | ribosomal protein L28e | 123.0(459) | 467.5(763) | 583.0(1306) |
| 135269_2 | ribosomal protein L11e | 48.8(263) | 137.8(325) | 236.3(765) |
| 11670 | ribosomal protein L34e | 21.7(221) | 96.9(432) | 105.1(643) |
| 4052 | ribosomal protein L34e | 86.0(729) | 391.8(1453) | 428.3(2180) |
| 135451_2 | ribosomal protein L22e | 70.1(623) | 190.6(741) | 353.6(1887) |
| 135464_1 | ribosomal protein L8e | 63.0(797) | 92.7(513) | 322.1(2446) |
| 134926_2 | ribosomal protein L6e | 69.6(488) | 356.2(1093) | 358.3(1509) |
| 135453_1 | ribosomal protein S3e | 311.6(2067) | 940.1(2727) | 1609(6408) |
| 135002_2 | ribosomal protein S23e | 216.1(484) | 1040(1019) | 1120(1506) |
| 135996_1 | ribosomal protein S9e | 133.7(1527) | 321.8(1607) | 695.2(4765) |
| 135415_3 | ribosomal protein L21e | 148.9(2361) | 464.7(3223) | 776.8(7395) |
| 136006_1 | ribosomal protein S11e | 11.6(56) | 35.5(75) | 61.0(177) |
| 129683 | ribosomal protein L17e | 169.5(302) | 524.8(409) | 904.1(967) |
| 135434_1 | ribosomal protein L35e | 44.0(337) | 187.2(627) | 236.4(1087) |
| 4516 | ribosomal protein S20e | 137.4(1133) | 490.5(1769) | 750.8(3717) |
| 64431 | ribosomal protein S27a | 33.4(110) | 156.9(226) | 182.6(361) |
| 135536 | ribosomal protein L32 | 286.6(2032) | 1381(4285) | 1572(6693) |
| 18863 | ribosomal protein L3 | 316.7(3945) | 305.3(1663) | 1756(13133) |
| 135269_1 | ribosomal protein L11e | 224.8(1405) | 632.5(1729) | 1258(4723) |
| 135464_2 | ribosomal protein L8e | 129.7(1347) | 227.3(1032) | 736.9(4593) |
| 18824 | ribosomal protein L12e | 37.4(281) | 114.2(375) | 213.0(960) |
| 11003 | ribosomal protein L15 | 140.8(1557) | 467.0(2258) | 804.9(5341) |
| 134822_2 | ribosomal protein L18Ae | 353.0(1584) | 1212(2379) | 2043(5505) |
| 136014 | ribosomal protein L13Ae | 271.3(2303) | 896.6(3329) | 1595(8129) |
| 9356 | ribosomal protein, putative | 1.2(24) | 4.1(36) | 7.1(86) |
| 9017 | ribosomal protein S13e | 2.9(15) | 11.3(26) | 17.2(54) |
| 112200 | ribosomal protein L35e | 46.6(70) | 199.4(131) | 279.5(252) |
| 16277 | ribosomal protein L7Ae | 396.7(3298) | 653.3(2375) | 2430(12128) |
| 13704 | ribosomal protein L7e | 92.9(814) | 244.6(937) | 576.2(3030) |
| 135434_2 | ribosomal protein L35e | 9.4(82) | 16.0(61) | 58.8(307) |
| 135271_2 | ribosomal protein S2e | 225.8(2178) | 590.2(2490) | 1413(8186) |
| 134852_2 | ribosomal protein S7e | 340.1(1964) | 731.7(1848) | 2144(7433) |
| 135440_1 | ribosomal protein L18e/L15 | 128.1(822) | 448.9(1260) | 809.5(3119) |
| 135250_1 | ribosomal protein S8e | 306.1(2990) | 774.6(3309) | 1964(11518) |
| 136006_2 | ribosomal protein S17 | 40.1(407) | 125.8(559) | 264.7(1614) |
| 1291 | ribosomal protein S6e | 169.3(1682) | 375.4(1631) | 1127(6727) |
| 135567_3 | ribosomal protein S3Ae | 409.6(2962) | 952.2(3011) | 2767(12010) |
| 135503_2 | ribosomal protein S18e | 85.7(307) | 268.7(421) | 584.5(1257) |
| 135440_2 | ribosomal protein L18e/L15 | 14.8(90) | 63.7(169) | 101.6(370) |
| 136019_1 | ribosomal protein S8 | 162.8(1261) | 1233(4177) | 1123(5221) |
| 135373_1 | ribosomal protein S14e | 153.8(825) | 1027(2410) | 1062(3420) |
| 135567_1 | ribosomal protein S3Ae | 184.5(470) | 370.8(413) | 1293(1978) |
| 135634_2 | ribosomal protein S19e | 78.2(590) | 194.8(643) | 555.1(2515) |
| 14875 | ribosomal protein L18e/L15 | 3.1(23) | 12.8(41) | 23.0(101) |
| 6818 | ribosomal protein L13e | 94.0(773) | 309.4(1112) | 722.2(3563) |
| 135190_1 | ribosomal protein SAe | 37.8(491) | 101.0(573) | 302.5(2356) |
| 7204 | ribosomal protein L1p/L10e family, putative | 31.9(412) | 50.0(282) | 256.4(1986) |
| 10405 | ribosomal protein L18p/L5e | 207.0(367) | 408.9(317) | 1672(1780) |
| 64389 | ribosomal protein S27a | 1.3(5) | 7.5(13) | 10.5(25) |
| 135190_2 | ribosomal protein SAe | 3.6(29) | 12.5(44) | 43.2(209) |
| 62264 | ribosomal protein L28e family, putative | 10.6(125) | 30.4(157) | 163.9(1161) |
| 98671 | ribosomal protein L7e | 0.2(1) | 6.7(16) | 5.5(18) |
| **Lysosome protein** | | | | |
| 61244 | papain family cysteine protease | 18.5(312) | 7.1(52) | 157.2(1587) |
| 62233 | papain family cysteine protease | 141.0(1795) | 580.8(3232) | 696.2(5318) |
| 61798 | papain family cysteine protease | 25.4(288) | 29.8(148) | 6.3(43) |
| 64724 | eukaryotic aspartyl protease | 1.3(16) | 22.6(121) | 13.6(100) |
| 8771 | eukaryotic aspartyl protease | 252.5(3395) | 816.6(4801) | 837.7(6760) |
| 99204 | eukaryotic aspartyl protease | 1.8(23) | 29.9(166) | 19.4(148) |
| 59214 | serine carboxypeptidase | 7.9(115) | 76.4(486) | 72.9(637) |
| 2823 | serine carboxypeptidase | 8.1(141) | 15.1(114) | 48.8(507) |
| **TCA cycle** | | | | |
| 63207 | oxoglutarate dehydrogenase (succinyl-transferring), E1 component | 16.8(541) | 10.5(148) | 58.4(1131) |
| 18954 | malate dehydrogenase, NAD-dependent | 101.2(1270) | 330.6(1814) | 391.5(2949) |
| 135754_1 | succinate-CoA ligase, beta subunit | 56.7(949) | 123.8(906) | 171.0(1717) |
| 53902 | fumarate hydratase (fumerase) | 4.3(75) | 21.4(164) | 49.5(520) |
| 7998 | pyruvate carboxylase | 1.3(47) | 16.8(275) | 84.7(1897) |
| 123811 | pyruvate carboxylase | 167.3(6962) | 6.9(125) | 24.2(605) |
| 65354 | malate dehydrogenase, NAD-dependent | 0.2(1) | 5.4(14) | 1.1(4) |
| 135880_2 | aconitate hydratase 2 | 8.3(243) | 56.0(718) | 247.8(4361) |
| 3968 | succinate dehydrogenase/fumarate reductase transmembrane subunit | 58.3(549) | 252.9(1041) | 69.2(391) |
| 48239 | pyruvate dehydrogenase complex dihydrolipoamide acetyltransferase | 59.7(136) | 4.0(4) | 4.4(6) |
| 66830 | dihydrolipoyl dehydrogenase | 49.9(874) | 0.9(7) | 0.2(2) |
| 597 | pyruvate dehydrogenase E2 component, putative | 20.7(232) | 8.8(43) | 80.6(543) |
| 65643 | pyruvate dehydrogenase E2 component | 81.9(1077) | 7.0(40) | 9.1(72) |
| 14886 | pyruvate dehydrogenase complex dihydrolipoamide acetyltransferase | 28.3(236) | 170.0(620) | 46.5(233) |
| 59184 | isocitrate dehydrogenase, NADP-dependent | 22.1(414) | 8.4(69) | 76.3(859) |
| 135468_2 | monomeric isocitrate dehydrogenase | 0.5(2) | 32.1(58) | 26.6(66) |
