# Supplementary material for: Changes in gene expression of Prymnesium parvum induced by nitrogen and phosphorus limitation
Source: Front Microbiol. 2015 Jun 24;6:631. doi: 10.3389/fmicb.2015.00631 (PMC4478897; doi:10.3389/fmicb.2015.00631)
Supplement: Supplementary file 3 [file Image1.PDF]

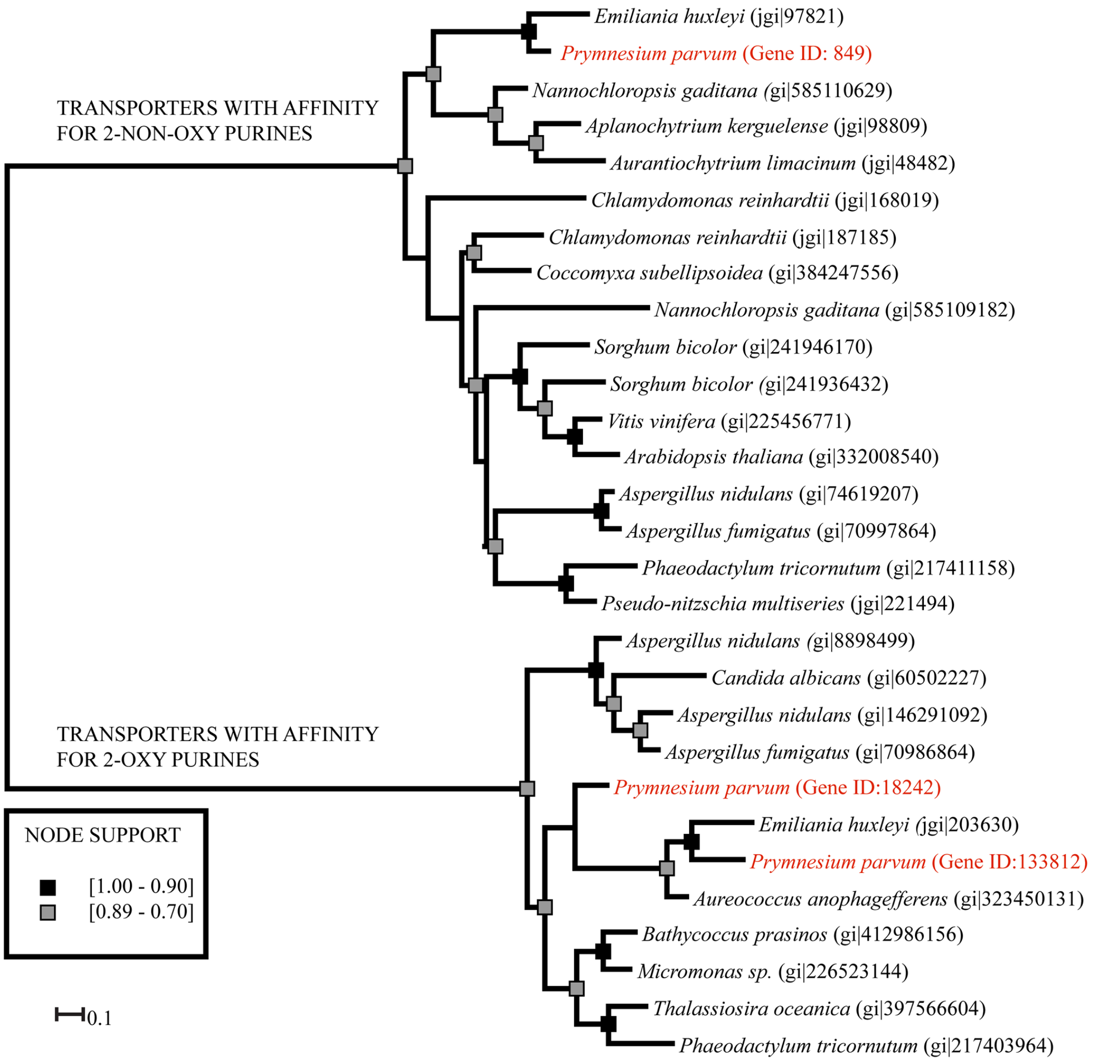

Supplemental Figure 1. Maximum likelihood phylogenetic tree of purine transporters obtained from the transcriptomes of *Prymnesium parvum* and other public database sequences. Each sequence is identified by the species name and its accession number (gi for sequences issued from GenBank and jgi for sequences obtained from JGI genomes). Separation between 2-non-oxy purine transporters and 2-oxy purine transporters are based on Terrado et al. (2015) and references within. Complete aligned sequences were filtered with GBlocks (Talavera and Castresana 2007), and the maximum likelihood trees were produced using Fasttree (Price et al. 2010) using an alignment of 149 positions. Bootstrap values at nodes are represented by black squares (support 1.00 – 0.90) and grey squares (support 0.89 – 0.70); support at nodes < 0.50 is not indicated.
