# Supplementary material for: Changes in gene expression of Prymnesium parvum induced by nitrogen and phosphorus limitation
Source: Front Microbiol. 2015 Jun 24;6:631. doi: 10.3389/fmicb.2015.00631 (PMC4478897; doi:10.3389/fmicb.2015.00631)
Supplement: Supplementary file 4 [file Image2.PDF]

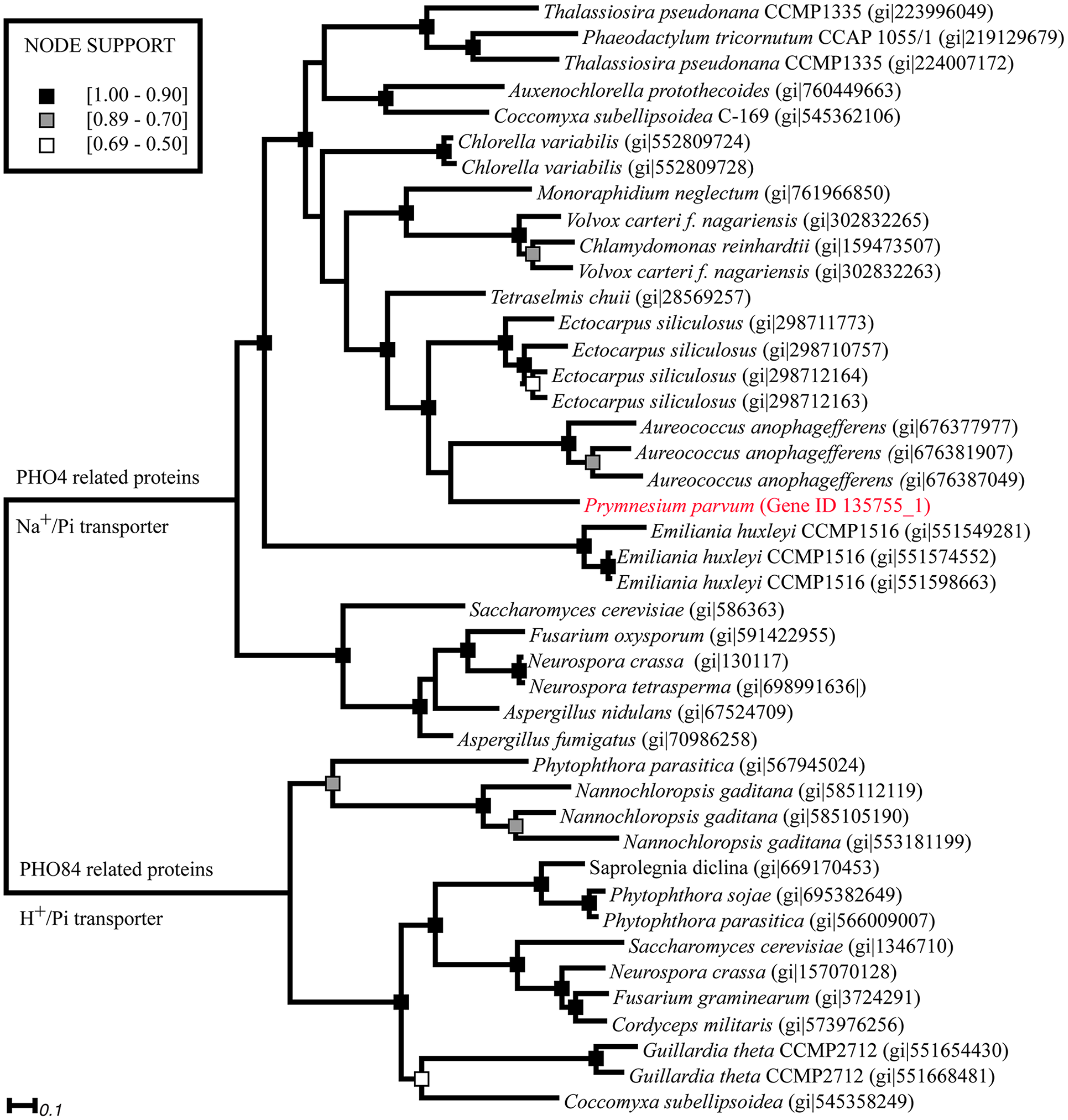

Supplemental Figure 2. Maximum likelihood phylogenetic tree of phosphate transporters obtained from the transcriptomes of *Prymnesium parvum* and other sequences available from GenBank (identified with its gi number). The tree presents the phylogenic relationship between PHO4 and PHO84, whose physiological functions have been experimentally established in other taxa (*i.e. Neurospra crassa* and *Tetraselmis chui*). Complete aligned sequences were filtered with GBlocks (Talavera and Castresana 2007) and the maximum likelihood trees were produced using Fasttree (Price et al. 2010) using an alignment of 150 positions. Bootstrap values at nodes are represented by black squares (support 1.00 – 0.90), grey squares (support 0.89 – 0.70) and white squares (support 0.69 – 0.50); support at nodes < 0.50 is not indicated.
